# Divalent metal transporter 1 (DMT1) in the brain: implications for a role in iron transport at the blood-brain barrier, and neuronal and glial pathology

**DOI:** 10.3389/fnmol.2015.00019

**Published:** 2015-06-08

**Authors:** Tina Skjørringe, Annette Burkhart, Kasper Bendix Johnsen, Torben Moos

**Affiliations:** Section of Neurobiology, Biomedicine, Institute of Medicine and Health Technology, Aalborg UniversityAalborg, Denmark

**Keywords:** iron, transferrin receptor, OX26, DMT1, blood brain barrier, Belgrade rat

## Abstract

Iron is required in a variety of essential processes in the body. In this review, we focus on iron transport in the brain and the role of the divalent metal transporter 1 (DMT1) vital for iron uptake in most cells. DMT1 locates to cellular membranes and endosomal membranes, where it is a key player in non-transferrin bound iron uptake and transferrin-bound iron uptake, respectively. Four isoforms of DMT1 exist, and their respective characteristics involve a complex cell-specific regulatory machinery all controlling iron transport across these membranes. This complexity reflects the fine balance required in iron homeostasis, as this metal is indispensable in many cell functions but highly toxic when appearing in excess. DMT1 expression in the brain is prominent in neurons. Of serious dispute is the expression of DMT1 in non-neuronal cells. Recent studies imply that DMT1 does exist in endosomes of brain capillary endothelial cells denoting the blood-brain barrier. This supports existing evidence that iron uptake at the BBB occurs by means of transferrin-receptor mediated endocytosis followed by detachment of iron from transferrin inside the acidic compartment of the endosome and DMT1-mediated pumping iron into the cytosol. The subsequent iron transport across the abluminal membrane into the brain likely occurs by ferroportin. The virtual absent expression of transferrin receptors and DMT1 in glial cells, i.e., astrocytes, microglia and oligodendrocytes, suggest that the steady state uptake of iron in glia is much lower than in neurons and/or other mechanisms for iron uptake in these cell types prevail.

## Introduction

Iron is essential for virtually all eukaryotic cells and indispensable for cellular processes such as DNA synthesis, mitosis, and metabolic processes that require synthesized proteins such as enzymatic reactions and electron transport in the respiratory chain (Hentze et al., 2010; Anderson and Vulpe, 2009; Rouault, 2013). Iron is involved in organ-specific functions e.g., in the brain, where it is involved in synthesis of neurotransmitters and myelin formation (Todorich et al., 2009; Rouault, 2013; Ward et al., 2014). Disruption of the iron status dramatically affects cellular functions, as both iron deficiency and excess compromise the cell. Consequently, a tightly regulated system evolved to ensure appropriate levels of available intracellular iron. Iron exists on two different ionic forms in biological systems, i.e., ferrous iron (Fe^2+^) and ferric iron (Fe^3+^; Anderson and Vulpe, 2009; Hentze et al., 2010; Rouault, 2013). Outside cells, iron appears on its oxidized, insoluble ferric form, and intracellularly, iron mainly stays on its reduced, but soluble ferrous form. Oxidation and incorporation by ferritin that also has ferroxidase activity prevents ferrous iron from accumulating unbound, which prevents iron from participating in unwanted oxidative reactions prone to generate oxidative stress and subsequent cell damage.

The cellular iron-regulatory system is complex and involves various proteins that collectively regulate import, neutralization, storage and export of iron. Functions that are mainly denoted by the transferrin receptor 1, divalent metal transporter 1 (DMT1), ferritin and ferroportin (Anderson and Vulpe, 2009; Rouault, 2013). The intracellular iron level is monitored by ubiquitously expressed iron-sulphur (Fe-S) cluster proteins, iron-responsive protein 2 (IRP2) in particular. In case of iron shortage, IRP2 loses its Fe-S cluster and binds to iron-responsive elements of target mRNAs. This binding of IRP2 stimulates translation of mRNA encoding proteins involved in cellular iron uptake (transferrin receptor 1 and DMT1) and post-transcriptionally represses the expression of proteins used for iron storage and export (ferritin and ferroportin) (Anderson and Vulpe, 2009; Hentze et al., 2010; Rouault, 2013).

Orally administered non-heme iron mainly enters the circulation via absorption by duodenal enterocytes in the gut. Ferric iron is reduced to ferrous iron by ferric reductase present on the apical surface of the enterocytes and subsequently transported across the apical cell membrane by DMT1 (Anderson and Vulpe, 2009; Hentze et al., 2010; Rouault, 2013). Conversely, heme-bound iron enters enterocytes by a mechanism not involving DMT1 but mediated by heme transporter carrier protein 1 (HCP1; Shayeghi et al., 2005). The iron-containing heme-complexes are degraded by heme-oxygenases, and the release of ferrous iron inside the enterocyte is subsequently exported into blood plasma by the iron-transporter, ferroportin (Anderson and Vulpe, 2009; Hentze et al., 2010; Rouault, 2013). Ferroportin operates in conjunction with the ferroxidases hephaestin and ceruloplasmin, which oxidize ferrous iron to ferric iron during the export from the enterocyte (Vulpe et al., 1999).

Once present in blood plasma, ferric iron is rapidly bound to apo-transferrin to form holo-transferrin, which binds two iron atoms. The iron uptake from holo-transferrin rely on the expression of transferrin receptor 1 present on the plasma membrane in many cell types, which ensures receptor-mediated endocytosis (Harding et al., 1983). The expression of transferrin receptor 1 is notably high on hepatocytes and neurons, the rapidly dividing cells of the erythron, and stem cells of the gut and skin (Rouault, 2013; Ward et al., 2014). Interestingly, endothelial cells of the vasculature with immediate access to holo-transferrin of the blood plasma do not contain detectable transferrin receptors, except for brain capillary endothelial cells that form the blood-brain barrier (BBB; Jefferies et al., 1984). The binding of holo-transferrin to the transferrin receptor quickly mediates uptake of the complex via clathrin-coated pits. The pH-value of the holo-transferrin/transferrin receptor complex-containing endosome drops to approximately 5.5 during the internalization process due to acidification promoted by an H^+^-ATPase proton pump in its membrane. The resulting acidic environment inside the endosome triggers the release of iron from transferrin (Dautry-Varsat et al., 1983). Furthermore, the ferrireductases like Steap3 reduces ferric iron to ferrous iron, and DMT1 pumps iron out of the endosome into the cytosol (Knutson, 2007). Transferrin receptor 1 recycles to the cell surface from where apo-transferrin is released into blood plasma ready for another iron-capture and intracellular iron-releasing cycle (Ciechanover et al., 1983; Dautry-Varsat et al., 1983).

The robust affinity of transferrin for iron is responsible for the complete absence of non-transferrin bound iron (NTBI) in normal conditions. However, in conditions with excess iron uptake from the gut or parenteral iron infusion, the iron-binding capacity of transferrin can be exceeded leaving to pathological deposition of iron widely in the body, a clinical condition called hemochromatosis (Ayonrinde et al., 2008; Brissot et al., 2011).

Transferrin receptor 2, located on hepatocytes, senses the saturation of extracellular, circulating transferrin, and a complex consisting of transferrin receptor 1, β_2_-microglobulin and hereditary hemochromatosis gene product located on membranes of dividing cells senses any excess intracellular iron and signals for an up-regulated synthesis and secretion of hepcidin (Chua et al., 2007; Worthen and Enns, 2014). The peptide hormone hepcidin is secreted from the liver and regulates body iron stores (Nemeth et al., 2004). Hepcidin has high affinity for ferroportin and post-translationally down-regulates ferroportin expression, thus hepcidin binding to ferroportin leads to its subsequent degradation, hereby lowering iron transport into the circulation (Nemeth et al., 2004).

## Outline

In this review, we evaluate the literature on uptake and transport of iron in the brain with a focus on delineating the possible roles of DMT1. The available literature reports on mutations in man, studies on normal and mutated rodent models, and various cellular models manipulating DMT1. We critically evaluate this literature and correlate the functions of DMT1 for iron uptake and transport in the brain with those published in non-neuronal tissues. As DMT1 is thought to play its significant function in concerted action with the transferrin receptor, we place special emphasis on the uptake and transport of iron in brain capillary endothelial cells and neurons, i.e., the two principal cell types with the highest expression of transferrin receptors in the brain (Moos et al., 1998, 2007). That neurons contain DMT1 is widely accepted, but the expression of DMT1 in brain capillary endothelial cells is a site of conflict, suggesting that iron could access the brain without involvement of DMT1. We include new data in this review that do signify a soft-spoken but significant expression of DMT1 for iron transport into the brain. The review also covers the role of DMT1 for iron uptake in neurons and non-neuronal cells of the brain, i.e., astrocytes, microglia and oligodendrocytes in health and pathology.

## DMT1 Protein Structure and Function

The DMT1 protein, also known as Nramp2, SLC11A2, and DCT1, conducts iron transport at two distinct compartments of the cell: (1) It facilitates iron uptake at the apical cell membrane in for instance duodenal enterocytes (Anderson and Vulpe, 2009; Hentze et al., 2010; Rouault, 2013); and (2) It transports iron across the endosomal membrane in almost all cell types that take up iron via the transferrin-transferrin receptor 1 pathway, e.g., erythroid precursors in the bone marrow (Canonne-Hergaux et al., 2001), and most cells in peripheral tissue. DMT1 functions optimally at an acidic pH of approximately 5.5, which fits well with the pH values in both the duodenum and inside the endosome.

DMT1 is a member of the natural resistance associated macrophage protein (Nramp) family. This highly conserved protein family consists of a varied group of membrane-bound divalent cation transporters (Cellier et al., 1995). Functional homologues of the Nramp proteins are found in bacteria (Makui et al., 2000; Agranoff et al., 2005). DMT1 transports divalent cations in exchange for one single proton (Gunshin et al., 1997). The substrate profile of DMT1 includes other metals than iron. For instance membrane-located 1A/(+IRE) DMT1 transports Fe^2+^, Cd^2+^, Co^2+^, Mn^2+^ (group 1), and Ni^2+^, VO^2+^, Pb^2+^ (group 2), but with decreasing efficiency, and group 1 being markedly more effectively transported than group 2. Zn^2+^ is not transported by DMT1 (Mackenzie et al., 2007). Other cations, such as Cu^1+^ and Cu^2+^ are also transported by DMT1 (Arredondo et al., 2003).

**Figure 1:**
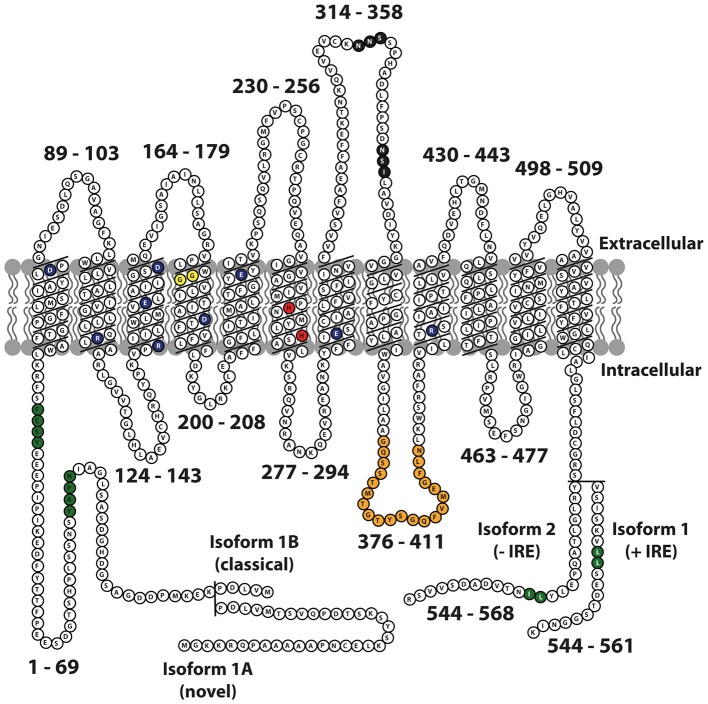
**Schematic representation of mouse Nramp2 (DMT1) isoform II (−IRE) and isoform I (+IRE) modified from Lam-Yuk-Tseung et al. (2003) by adding the IA and IB isoforms**. The 12 transmembrane domains and 13 Individual predicted intracellular and extracellular segments are identified, and their position within the primary sequence is shown. Amino acid residues defining sequence landmarks and signature motifs are depicted in different colors, including negatively and positively charged residues within predicted TM domains (dark blue), conserved histidine residues in TM6 (red), glycine residues in TM4 altered in anemic mk/Belgrade mutants (Gly185Arg), and mutated (in Nramp1) in mice susceptible to infections (Gly184Asp) (yellow). Also identified are Asn-linked glycosylation signals in the TM7-TM8 extracytoplasmic loop (black), predicted membrane targeting/sorting motifs (tyrosine-based and dileucine) (green), and consensus transport signature common to Nramp orthologs and present in the cytoplasmic face of membrane anchors of bacterial periplasmic permeases (orange). The two different C-termini of the protein generated by alternative mRNA splicing containing or not an iron response element (isoform I, +IRE; isoform II, IRE) in the 3′ untranslated region are identified, with corresponding numbering. Adapted from Lam-Yuk-Tseung et al. (2003).

The DMT1 protein has 12 transmembrane domains, membrane targeting motifs, one consensus transport motif, and two asparagine-linked glycosylation signals in an extra cytoplasmic loop (Figure 1). Both the N- and C-terminus of the protein are located in the cytoplasm. Several studies have elucidated the structure-function relationship of Nramp H^+^-coupled divalent metal transport: The transmembrane domain 1 (TMD1) and TMD6 are crucial for Nramp symport of metal ions and H^+^ ions (Chaloupka et al., 2005).

DMT1 was first identified in the mouse in 1995 (Gruenheid et al., 1995) and later cloned from a cDNA library prepared from duodenal mRNA in rats fed a low iron diet (Gunshin et al., 1997). The DMT1 gene comprises 17 exons and spans more than 36 kb. There are two alternative transcripts of the 3′ UTR, which were later identified as one with an iron response element (+IRE; Type 1) and one without an IRE (−IRE; Type 2; Tchernitchko et al., 2002). The IRE is a conserved stem loop structure, which by binding specific iron-response proteins in the cell mediates modified stability or translation efficiency of the given mRNA. Two additional transcripts have DMT1 differing in the 5′ region: One mRNA transcript starts in exon 1A, skips the next exon (1B), and splices directly with exon 2. Another mRNA transcript, the first identified, skips exon 1A, starts in exon 1B and splices with exon 2 (Hubert and Hentze, 2002). Exon 1A has an initiation codon (AUG), and variant 1A has a 29–31 amino acid extension in contrast to variant 1B, where the initiation codon is located in exon 2. The four identified DMT1 isoforms, 1A/(+IRE), 1A/(−IRE), 1B/(+IRE), and 1B/(−IRE) share 543 amino acid residues (Figure 1). The 3′ end of the IRE variant is unique for each variant and consists of 25 (−IRE) and 18 (+IRE) amino acids, respectively. A miRNA target sequence for let-7d is present in the 3′UTR of the (–IRE) DMT1 isoforms. Let-7d binds and participates in regulating the (–IRE) DMT1 isoform in erythroid cells (Andolfo et al., 2010). Differences occur in translational efficiency between the four isoforms (Mackenzie et al., 2007), and protein degradation pathways vary between the IRE isoforms. 1B isoforms are degraded by the ubiquitin E3-ligase parkin, whereas the 1A isoforms are not (Garrick et al., 2012).

The four DMT1 isoforms exhibit different organ-specific expression patterns. The 1A transcripts occur almost exclusively in polarized cells in duodenum and kidney, whereas the expression of 1B transcripts is ubiquitous. The expression of both of the IRE variants is to some extent found in most tissues. Therefore, the organ specific expression of DMT1 seems to depend on elements in the promoter of the 1A variant or in the 1A region itself (Hubert and Hentze, 2002). The subcellular distribution of the four isoforms varies as shown by anti-DMT1 immunocytochemistry on Xenopus oocytes transfected with each of the four DMT1 variants, respectively. The (+IRE) variants are localized to the cell membrane, whereas the (−IRE) variants are not (Mackenzie et al., 2007). The fact that there are four different isoforms of DMT1 might also reflect various functional properties. Importantly, however, the four isoforms transport Fe^2+^ at the same turnover rate and exhibit no differences in their functional properties, permeant ions or rate limiting steps (Mackenzie et al., 2007). Other studies have also failed to detect functional differences of the various isoforms (Garrick et al., 2006). Hence, the existence of the four isoforms is suggested to meet the need of cell type specific subcellular targeting and regulation (Mackenzie et al., 2007).

## Iron Regulates DMT1 Expression both Transcriptionally and Post-Transcriptionally in Non-Neuronal Cells

Papers published before 2002 report on both DMT1 IRE isoforms, but do not take the structure of the 5′ end of the DMT1 mRNA into consideration. This may affect the interpretation of data, as results may reflect effects of either of the 1A and 1B isoforms. A thorough study from 2002 elucidated the gene expression and transcriptional regulation of all four isoforms of DMT1 with respect to changes in iron levels (Hubert and Hentze, 2002). The study included semi-quantitative PCR on Caco-2 cells, and on mouse duodenum and kidney, using primers specific for each of the four isoforms. The results showed that the 1A region is the main player with respect to iron-dependent regulation of DMT1 expression in Caco-2 cells and duodenum. The 3′UTR with the IRE element also contributed to iron-responsive regulation, but to a lesser extent (Hubert and Hentze, 2002). Furthermore, the DMT1 IRE element has no effect on DMT1 expression in the colon cell line HT-29 transfected with a plasmid encoding a reporter with the (+IRE) 3′UTR of DMT1 (Tchernitchko et al., 2002). These results describe DMT1 mRNA data but additional levels of regulation may affect the protein, e.g., protein relocation in response to excess iron occurs in intestinal Caco-2 cells, which normally express high levels of cell membrane-bound DMT1. In Caco-2 cells subjected to high levels of non-heme iron, the level of cell membrane-bound DMT1 decreases without parallel changes in total mRNA levels, including both IRE isoforms. This relocation may be a rapid response regulatory mechanism for immediate response to changes in iron levels (Sharp et al., 2002).

## Mutations in the DMT 1 Gene Affect Gastrointestinal Iron Absorption, and Tissue Iron Deposition

DMT1 is significantly implicated in gastrointestinal absorption of iron as demonstrated in two animal models: the microcytic anemia (*mk*) mouse and in the Belgrade rat. Both of these rodents have a spontaneous G185R missense mutation in DMT1 located in the conserved TMD4 region (Figure 1) irrespective of isoform that causes defective DMT1 protein expression without complete loss of function (Gunshin et al., 2005). The DMT1 protein carrying the G185R mutation is abnormally glycosylated, less stable, and 20–35-fold less active in metal transport (Fleming et al., 1998; Su et al., 1998; Canonne-Hergaux et al., 2001). The Belgrade rat displays a phenotype with anemia. It has liver iron loading, possibly due to due impaired erythropoiesis (Thompson et al., 2006). It also has a lower concentration of iron in the brain (Farcich and Morgan, 1992; Burdo et al., 1999). The *mk* mouse is poorly viable although able to survive for more than a year (Xu et al., 2004).

Dietary iron-supplementation leads to increased hematocrit in the Belgrade rat (*b/b*; Thompson et al., 2006). Although the increase is lower than in age-matched heterozygotes (*b/+*), Belgrade rats absorb some intestinal iron despite defects in DMT1 function. A murine DMT1 knockout model shows a more severe phenotype than seen in the animals with the G185R missense mutation (Gunshin et al., 2005). Pups only survive for 1 week after birth, probably due to severe anemia, as the iron stores rapidly deplete, which confirms that DMT1 is the major intestinal iron transporter for non-heme iron from the diet in these murine models. The G185R mutation, reported independently at least three times in rodents, is remarkable. This coincidence suggests a gain of function of the DMT1 protein carrying the G185R mutation (Xu et al., 2004).

Rare cases of DMT1 mutations in humans occur, although it is a rare and under-diagnosed condition with an estimated prevalence of less than 1:1.000.000 (Mims et al., 2005; Beaumont et al., 2006; Iolascon et al., 2006; Blanco et al., 2009; Bardou-Jacquet et al., 2011)^1^. In contrast to rodents in where heme iron is poorly absorbed, one third of dietary iron in humans derives from heme iron and therefore bypasses the DMT1 route of the duodenal uptake (Conrad and Umbreit, 2000). Accordingly, mutations in DMT1 in man will primarily affect cellular iron uptake and utilization more than iron absorption, which leads to a somewhat different phenotype than the one seen in the rodents. The literature reports on five mutations in the human DMT1 gene, i.e., a 1285G>C mutation: The patient is homozygous for the mutation, which results in both a conservative amino acid substitution (E399D) and a preferential, but incomplete, skipping of exon 12 during RNA processing (Lam-Yuk-Tseung et al., 2005; Mims et al., 2005). A missense mutation (1246C>T, R416C) located in a conserved sequence in TMD9 and an additional 3bp deletion in intron 4 (c.310-3_5del) leading to aberrant mRNA splicing of exon 5 (Iolascon et al., 2006; Lam-Yuk-Tseung et al., 2006). A 3 bp exonic deletion in exon 5 removing residue V114, and a missense mutation in exon 8 leading to the exchange of a conserved amino acid (G212V), which in turn leads to changes in TMD2 and TMD5, respectively (Beaumont et al., 2006). A fourth patient has a N491S mutation and a G212V mutation. The N419S mutation results in disturbed protein trafficking, and the protein ends up in the endoplasmic reticulum (Bardou-Jacquet et al., 2011). The clinical phenotypes of these patients are, like in the Belgrade rat, microcytic anemia and liver iron overload, but none reports on pathological levels of iron in the brain. A fifth patient who is homozygous for the mutation G75R located in TMD1, displays a partly different phenotype, as no liver iron overload is observed (Blanco et al., 2009). This patient was only 7 years old at the time of publication, and the authors state that a possible later development of iron overload is possible.

The iron transfer between the mother and the fetus occurs via transferrin receptors expressed at the apical membrane of the placental cells (Cetin et al., 2012). However, the normal transferrin-transferrin receptor 1 route is apparently not exclusive, as demonstrated by studies in DMT1 knockout mice in where neonatal hematological parameters are normal (Gunshin et al., 2005). Moreover, the liver also seems to have an alternative route for iron uptake in the fetus, as liver iron stores are large and even elevated immediately after birth in the DMT1 knockout mice as compared to wild type mice (Gunshin et al., 2005). The iron overload in the liver in animal models and patients with DMT1 mutations may therefore be due to iron uptake through a DMT1-independent route and a subsequent disruption of the DMT1-mediated iron delivery essential to hemoglobin production for erythropoiesis (Thompson et al., 2006).

## The Expression of DMT1 in Brain

The gene expression of the DMT1 in the brain presumably solely relies on the 1B isoform of the N-terminal that is expressed together with both IRE isoforms (+/− IRE) in the mouse brain (Hubert and Hentze, 2002). Recent studies confirm that the brain expresses the two IRE isoforms of the C-terminal (Ke et al., 2005), and that the 1A is not expressed in neurons of the rat brain (Pelizzoni et al., 2012). At the protein level, the consensus is less clear. The DMT1 is present in the brain when examined using two different antibodies that detect the conserved regions (Moos et al., 2000; Burdo et al., 2001). DMT1 is also detectable using an antibody that detects the IRE isoforms (Moos and Morgan, 2004). The detection of immunoreactivity of the 1A form using a polyclonal antibody is more questionable given the data from the gene expression analyses (Hubert and Hentze, 2002) and may rely on cross-reactivity between the DMT1 isoforms. The scarce expression of the IRE isoforms in the developing brain follows with an increase during the first postnatal weeks (Moos and Morgan, 2004; Ke et al., 2005; Moos et al., 2006). The cellular distribution of DMT1-immunoreactive cells in the brain remains controversial. Clearly, consensus points towards a sustained expression in neurons, whereas studies report on varying protein expression in DMT1 in non-neuronal cells: Astrocytes, microglia and oligodendrocytes, and the two principal cell types that form the brain barriers to molecules of the blood, i.e., brain capillary endothelial cells and choroid plexus epithelial cells. The virtual absent expression of transferrin receptors and DMT1 *in vivo* in astrocytes, microglia and oligodendrocytes suggests that the steady state uptake of iron in glia in physiological conditions is much lower than in neurons and/or other mechanisms for iron uptake in these cell types prevail (see below).

## DMT1 has a Role for Iron Transport into the Brain

**Figure 2:**
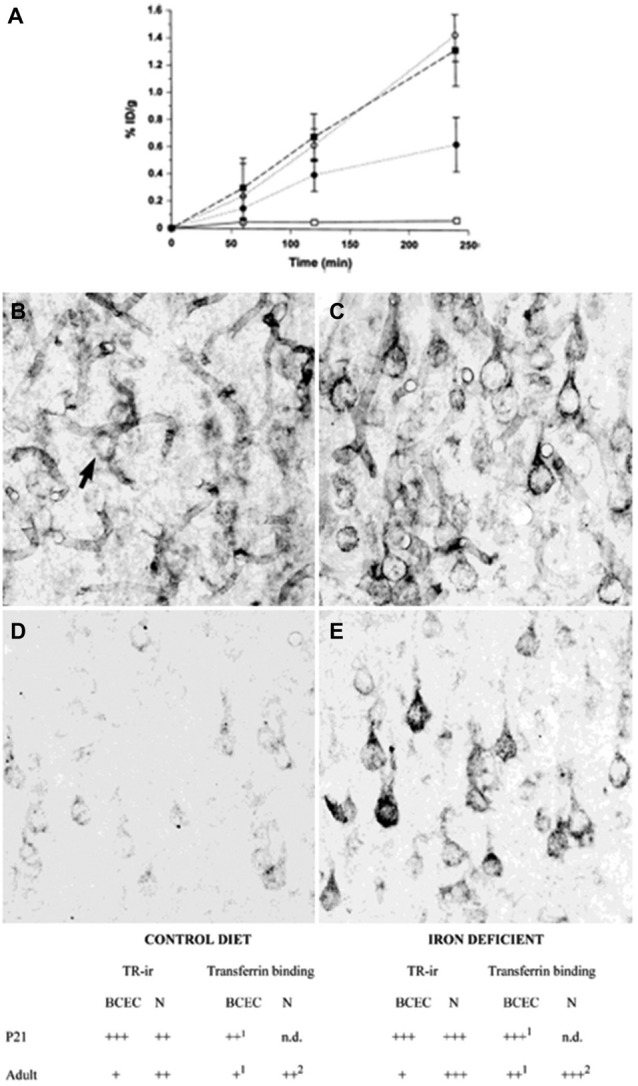
**Transferrin receptors in the normal and iron-deficient developing and adult brain. (A)** Binding of a monoclonal antibody to the transferrin receptor (OX26) in the developing rat brain. The binding is expressed as % ID/g weight ± SD. The uptake of OX26 is age-dependent being highest in P15 brains. Iron-deficiency does not affect the uptake of OX26 in injected P15 rats. □, adult rat; ▪, P15 normal rat; ○, P15 iron-deficient rat; ● P0 rat (Moos and Morgan, 2001). **(B–E)** Transferrin receptor immunoreactivity (TR-ir) in the pyramidal cell layer (layer V) of the neocortex from P21 **(B,C)**, and adult rat brain **(D,E)**. In the young (P21) rat brain **(B)**, brain capillaries and neurons (arrow) display TR-ir. When subjected to iron deficiency neuronal TR-ir is elevated in the P21 brain **(C)**. In the normal adult rat brain **(D)**, TR-ir is seen in pyramidal neurons. The TR-ir in brain capillaries is much less pronounced than at P21. Following iron deficiency, a higher TR-ir is seen in pyramidal neurons of the iron deficient rat **(E)** without changes in the capillary TR-ir. Scale bars 20 μm. **Below**. Summary of transferrin receptor immunoreactivity (TR-ir) and transferrin binding in brain capillary endothelial cells (BCEC) and neurons (N) of control and iron deficient rats at different stages of development. The levels of TR-ir and transferrin binding were evaluated in four degrees: +++, very strong, ++, strong, + weak, −, absent. nd, Not determined. Levels of transferrin binding was determined by *in vivo* uptake (Taylor and Morgan, 1990; Taylor et al., 1991; Crowe and Morgan, 1992) and *in vitro* receptor autoradiography (Moos et al., 1998). Adapted from Moos et al. (1998) and Moos and Morgan (2001).

The transport of iron across the endothelial cell layer of the BBB is still not fully explained. The main inducer of iron uptake is the binding of circulating holo-transferrin to transferrin receptor 1 expressed on the luminal side of the brain capillary endothelial cells (c.f. Rouault and Cooperman, 2006; Moos et al., 2007). This capillary expression is specific for the brain and spinal cord and exerts a pattern where approximately 10% of the transferrin receptors can be detected on the cell surface, whereas the remaining 90% are presumably located as a spare pool inside the cytoplasm (van Gelder et al., 1995; Visser et al., 2004). Brain iron deficiency is accompanied by an increase in iron uptake across the BBB, but interestingly this is not due to an increased expression of brain endothelial transferrin receptors (Figure 2) (Moos et al., 1998). Instead, the increased iron uptake under these conditions is due to a raise in the cycling rate of transferrin receptors (Moos and Morgan, 2001; Moos et al., 2007).

Subsequent to the transferrin receptor-mediated binding and resulting uptake of holo-transferrin at the BBB, the iron bound to transferrin has to traverse the cell to undergo release on the abluminal side into the brain interstitium. The mechanism for this movement, however, is highly debated, but two main hypotheses stand out: (1) Receptor-mediated transcytosis leading to release of holo-transferrin inside the brain interstitium; and (2) Receptor-mediated endocytosis of transferrin followed by reduction of iron inside endosomes and retro-endocytosis of apo-transferrin to the luminal surface. Fe^2+^ is pumped out of the endosome into the cytosol by DMT1 and transported to the abluminal side, where facilitated transfer occurs across the abluminal membrane by ferroportin followed by re-oxidation to Fe^3+^ due to the ferroxidase activity of ceruloplasmin (Moos et al., 2007).

Concerning the first hypothesis (Hypothesis I), there is no evident quantitative transport of transferrin through the BBB (Crowe and Morgan, 1992; Strahan et al., 1992; Moos et al., 2006), which we have covered in previous reviews on brain capillary endothelial cells (Moos and Morgan, 2000; Moos et al., 2007). The bulk of iron taken up by brain capillary endothelial cells undoubtedly undergoes transport further into the brain, but there is no evidence of a similar transport of transferrin. Noteworthy, transferrin remains un-degraded within brain endothelial cells, which could otherwise be a plausible explanation for the much higher uptake of iron than of transferrin (Strahan et al., 1992). Intravenously injected [^59^Fe-^125^I] transferrin leads to formation of non-transferrin-bound ^59^Fe in the brain, which also favors hypothesis I on iron being detached from transferrin within the brain endothelial cells (Moos et al., 2007). Newer studies of antibody-mediated drug delivery to the brain via the transferrin receptor suggest that modification of the antibody affinity towards mimicking that of endogenous transferrin may induce transcytosis (Yu et al., 2011; Niewoehner et al., 2014; Pardridge, 2015). This could rely on a mechanism where the lower affinity for the transferrin receptor leads to detachment from the receptor inside transcytotic vesicles, followed by release into the brain interstitium by non-specific exocytosis. Counteracting this observation, injection of holo-transferrin with high affinity for the rat transferrin receptor in young rats with high transferrin receptor expression at the BBB is not accompanied by transcytosis (Moos et al., 2006).

**Figure 3:**
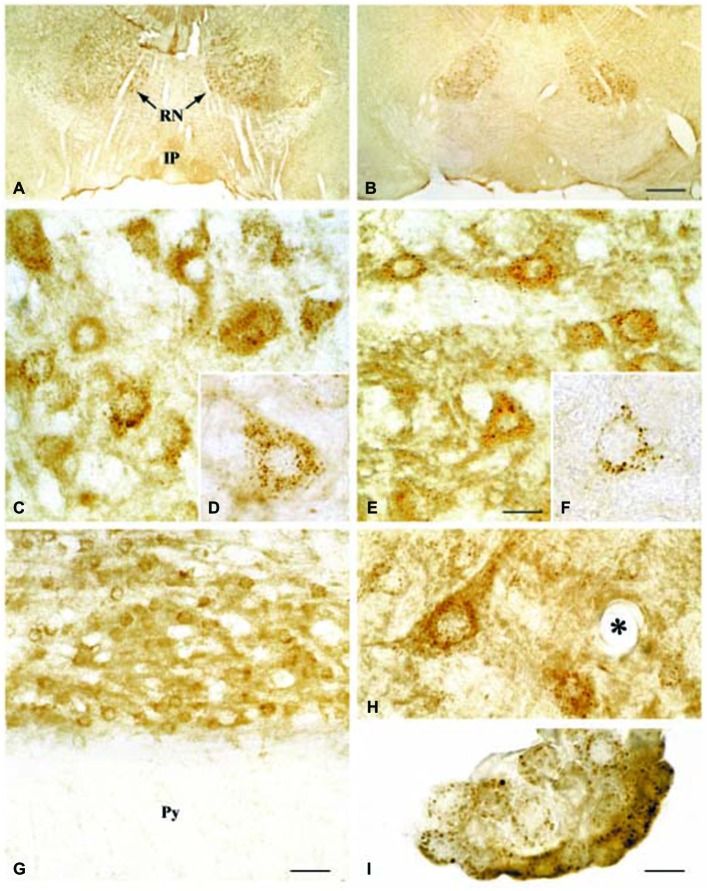
**Expression of DMT1 in Wistar (A,C,D,G,H,I) and b/b (B,E,F) rat brains. (A,B)** Sections from the mesencephalon containing the red nucleus (RN) and the interpedunculate nucleus (IP) shown at low power magnification. The DMT1 expression is not higher in the b/b brain.** (C,E)** Sections showing the RN at medium power. DMT1 immunoreactivity is seen in neurons of both rat strains. The intensity of the neuronal DMT1 immunoreactivity does not differ between the two rat strains. **(D,F)** Labeled neurons from the RN shown at high power. Note the punctuated immunoreaction product that distinctly labels the perikarya and leaves the nucleus unstained. **(G)** Section from the pyramis of the brainstem. Whereas brainstem neurons of the reticular formation (top) are consistently labeled, elements of the neighboring white matter (wm) are virtually unstained. **(H)** High contrast image taken from the lower brainstem shown at high power. Elements with morphology corresponding to transected brain capillaries are unlabeled (the asterisk identifies a transected capillary). A labeled neuron is also seen. **(I)** DMT1 in choroid plexus epithelial cells of the third ventricle. Scale bars: A,B = 175 μm; C,E = 20 μm, D,F,H,I = 10 μm, G = 30 μm. Illustrations partly adapted from Moos and Morgan (2004).

**Figure 4:**
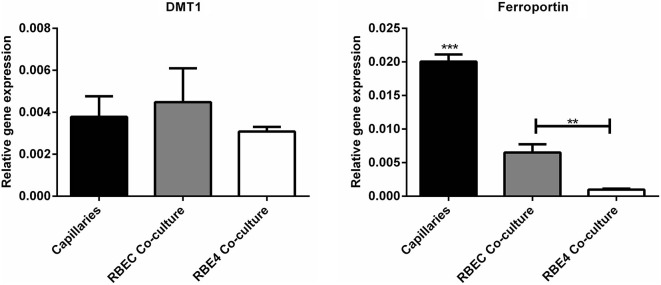
**Gene expression analyses for molecules related to iron uptake *in vivo* and in BBB cultures. (Left)** Divalent metal transporter I (DMT1). **(Right)** Ferroportin. Gene expression was analyzed in purified brain capillaries from 21 days old rats, which were subsequently cultured in primary co-culture with astrocytes to enable a confined BBB *in vitro* rat brain endothelial cells (RBEC). The expression was compared with an immortalized cell line, RBE4. The expression of both DMT1 and ferroportin is present in both purified capillaries and primary cultures. Data are normalized to β-actin and presented as means ± SEM (*n* = 6). **p < 0.01, ***p < 0.001 (Burkhart, 2015).

**Figure 5:**
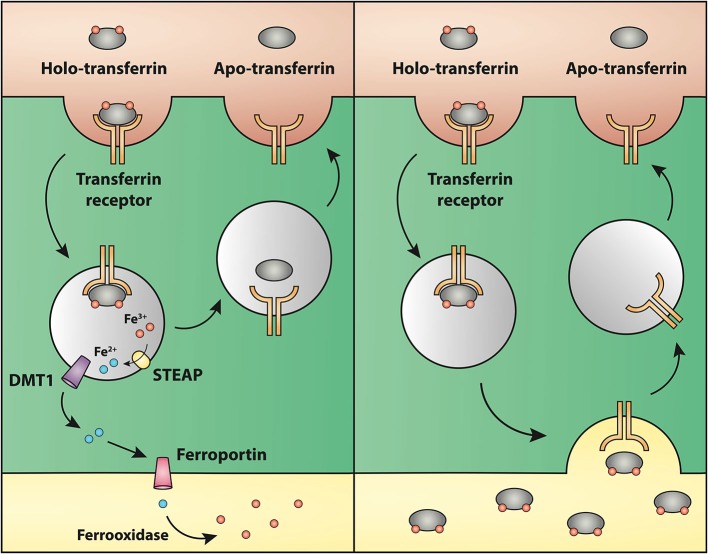
**Drawing showing a model proposing the transport of iron through the blood-brain barrier without (left) or with (right) transcytosis of transferrin. (Left)** The recent identifications of Steap, DMT1 and ferroportin in carefully purified brain capillaries strongly favor that iron gets detached from transferrin inside endosomes, which is then followed by iron efflux into the brain interstitium mediated by ferroportin. This is the model that is referred to Hypothesis I in the text. **(Right)** drawing showing the claimed transcytosis of holo-transferin through the brain capillary endothelial cell, which in the text is referred to as Hypothesis II.

Regarding the second hypothesis (Hypothesis II) in an effort to explain the apparent absences of DMT1 and ferroportin, it was hypothesized that iron would be transported through the brain endothelial cells without the involvement of an endosomal escape mediated by DMT1 with little or no iron being pumped out of the trafficking vesicles into the cytosol (Moos et al., 2007). Moreover, according to this proposed theory, the vesicle could dock at the abluminal surface of the brain capillary endothelial cells where, facilitated by extracellular factors present in the local abluminal microenvironment, iron could detach from transferrin mediated by a decrease in the extracellular pH and be released in the brain interstitium. Such factors could derive from astrocytic release and might include ATP, hydrogen ions, nucleotides and citrate (Morgan, 1977, 1979). Favoring the influence of astrocytes, they form end-feet with intimate contact with the brain capillaries and cover approximately 95% of their basal lamina (Brightman and Reese, 1969; Kacem et al., 1998; Abbott et al., 2006). Moreover, the distribution and transport of iron within brain endothelial cells is dramatically different in the developing rat brain at the time point where astrocytes have not yet formed contact with the brain capillaries (Moos et al., 2006). Declining the appraisal of hypothesis II, examination of the *in vivo* expression of DMT1 revealed an abundant expression in neurons but not at detectable levels in brain endothelial cells in spite using high sensitivity immunoassays (Figure 3) targeting different epitopes on DMT1 (Moos et al., 2006). Gene expression analysis failed to confer DMT1 among transporters in isolated rat brain endothelial cells capillaries (Enerson and Drewes, 2006), which confer earlier observations failing to DMT1 mRNA in endothelial cells of the mouse brain (Gunshin et al., 1997). On the contrary, we have recently demonstrated a quite low expression of both DMT1 and ferroportin mRNA and protein in primary culture of rat brain endothelial cells with confined BBB properties (Figure 4; Burkhart, 2015). Therefore, these results indicate that the expression of DMT1 in brain endothelial cells is low. Supporting the latter, studies in the homozygous Belgrade b/b rats revealed that uptake of iron in isolated brain capillaries was lower, but also that the major quantitative difference in iron uptake between heterozygous Belgrade b/+ occurred in the postvascular compartment containing the neurons that are strong in DMT1 expression (Moos et al., 2006). Furthermore, short-term perfusion studies (5 min) performed in young Belgrade rats showed that the percentage of iron present in the brain capillary fraction was lower, albeit only slightly, in the Belgrade rat than in controls (Moos et al., 2006). We believe our recent identification of DMT1 and ferroportin in carefully purified brain capillaries strongly favor the notion of detachment of iron from transferrin inside endosomes followed by efflux into the brain interstitium (Figure 5). In support of this notion, recent studies also detected ferroportin and ferroxidases in cultured brain endothelial cells (McCarthy and Kosman, 2013; McCarthy et al., 2014). Probably, the presumably low expression of DMT1 in the brain endothelial cells is sufficient to balance the iron entering endosomes subsequent to receptor-mediated internalization of transferrin, hence supporting hypothesis II.

A recent study suggests that brain endothelial cells should be regarded as more than a simple conduit where iron is merely transported through, but as a reservoir of great importance in the regulation of brain iron homeostasis via signaling from the brain interior to regulate the trafficking of the transferrin receptors cycling (Simpson et al., 2015). The iron status within the brain interstitium may well be reflected in the percentage of transferrin saturation with iron and probably also the presence of non-transferrin bound iron. As a prerequisite for the very original consideration on regulated iron transport from the brain endothelium and further into the brain proposed by Simpson et al. (2015). The brain endothelium needs to express transferrin receptors at its abluminal side to sense the transferrin saturation within the brain, but so far the detection of transferrin receptors there is limited and without consensus. However, the presence of non-transferrin bound iron may enter the brain endothelial cells from the brain interior and thereby affect the recycling of the transferrin receptor.

## Uptake of Non-Transferrin Bound Iron at the Blood-Brain Barrier

Whether circulatory iron excess leads to iron deposition in the brain in hemochromatosis is site of dispute. Recent studies imply that hereditary hemochromatosis can affect the handling and acquisition of iron with neurons and glia (Bartzokis et al., 2011; Acikyol et al., 2013). Concerning blood to brain transport of iron, rodents with experimentally induced hemochromatosis do not accumulate iron in the brain (Moos et al., 2000). There is no evidence for uptake and transport of non-transferrin bound iron at the BBB even under conditions with experimental hemochromatosis where the iron-binding capacity of transferrin is exceeded (Kim et al., 2013). Theoretically, expression of the 1A isoform of DMT1 in the luminal membrane of brain capillary endothelial cells could allow circulatory non-transferrin bound iron to enter brain. However, as previously stated there is no solid evidence for expression of the 1A isoform in these cells.

## The Participation of DMT1 in Neuronal Iron Uptake

In contrast to brain capillaries, neurons upregulate their expression of the transferrin receptor and transferrin binding in iron deficiency (Moos et al., 1998). Neurons do not increase their expression of the DMT1 IRE isoforms in iron depleted conditions (Ke et al., 2005). The neuronal DMT1 seems to co-localize with transferrin-receptor containing endosomes and probably participates in the uptake of iron subsequent to the binding and internalization of holo-transferrin to the neuronal transferrin receptor (Moos et al., 2007; Rouault, 2013; Ward et al., 2014). Hence, there is good reason to believe that neurons acquire iron by the well-described, mechanism occurring in cells outside the brain where transferrin receptor-mediated internalization leads to iron transport to the endosome and retro-endocytosis of apo-transferrin and transferrin receptor-containing coated pits to the neuronal surface. Iron is then subsequently reduced by ferrireductases inside endosomes, pumped into the cytosol by DMT1 to participate in the neurons’ metabolism if not stored in ferritin or transported out of the cell by ferroportin (Moos et al., 2007; Rouault, 2013; Ward et al., 2014).

Neurons take up non-transferrin bound iron *in vitro* (Pelizzoni et al., 2012; Ji and Kosman, 2015), and the presence non-transferrin bound iron in the intact brain (Moos and Morgan, 1998) suggests that this uptake probably also occurs *in vivo*. Concerning a role for DMT1 for non-transferrin bound uptake *in vivo* the evidence is unclear. Probably, such uptake would require the expression of the 1A isoform in the cellular membrane of neurons but this is not the case (Pelizzoni et al., 2012). Interestingly, however, transfection of the 1A form into primary hippocampal neurons provides the requisite for uptake of non-transferrin bound iron (Pelizzoni et al., 2012).

The role of DMT1 for neuronal iron handling in pathological studies is vaguely studied, but some interesting data have occurred. Mice mutated in DMT1 better overcome the toxic insult than control littermates in an experimental model for Parkinson’s disease, probably because the tendency towards iron accumulation is reduced (Salazar et al., 2008). Mutated Parkin is a cause of early onset of Parkinson’s disease, and Parkinson’s disease patients with mutated Parkin have higher levels of 1B/(+IRE) DMT1, reflecting the impairment of the ubiquitin E3-ligase activity (Roth et al., 2010). Reduction of another ubiquitin ligase Nedd4 family-interacting protein 1 (Ndfip1) could contribute to neurodegeneration via dysregulation of its regulation of DMT1 degradation (Howitt et al., 2014; Jia et al., 2015). Possibly this could lead to increased neuronal iron uptake and propagate iron-mediated oxidative stress and damage. The expression of both 1B/(−IRE) DMT1 and intracellular iron influx are increased in brain ischemia (Ingrassia et al., 2012). DMT1 +IRE is downregulated in 6-hydroxydopamine intoxicated, cultured dopaminergic neurons treated with brain-derived neurotrophic factor (BDNF) and glial cell line-derived neurotrophic factor (GDNF) (Zhang et al., 2014), but elevated following treatment with angiotensin (Garrido-Gil et al., 2013).

### DMT1 is Virtually Absent from Glial Cells *In Vivo* but Contributes to Iron Uptake in Astrocytes *In Vitro*

There is solid evidence for DMT1 expression in astrocytes *in vitro* but the *in vivo* evidence is more scarce (Burdo et al., 2001; Lis et al., 2004; Moos and Morgan, 2004; Huang et al., 2006; Pelizzoni et al., 2012). Interestingly, astrocytes do not express detectable levels of transferrin receptors *in vivo* but clearly *in vitro* and in primary rat astrocytes (Qian et al., 1999; Lis et al., 2004; Pelizzoni et al., 2012). The amount of available iron relative to the iron need regulates the expression of both transferrin receptor 1 and DMT1, and the differences between *in vivo* and *in vitro* conditions could possibly be attributed to the much higher proliferating state of cultured cells *in vitro*.

Western blot analyses of DMT1 purified from neurons and astrocytes show that the molecular mass of DMT1 in the two cell types differs (Pelizzoni et al., 2012). The DMT1 from neurons produces a band corresponding to the expected ~64 kDa, whereas astrocytes produce one of slower mobility. The size of the band obtained from astrocytes corresponds to those obtained from duodenal enterocytes. In astrocytes treated with an N-glycosylation-inhibitor, the DMT1 bands from neurons and astrocytes are both ~50 kDa, suggesting that DMT1 is differentially glycosylated in the two types of brain cells. As the same pattern of glycosylation exists for DMT1 in enterocytes known to acquiesce NTBI, a subcellular localisation with an extracellular presentation of DMT1 could confer transferrin-independent iron uptake across the cell membrane.

Cultured astrocytes seem to harbor distinct routes through which NTBI enters the cells. One route mediates the uptake of ferrous iron and depends on DMT1 and ascorbate (Lane et al., 2010). Ascorbate released from the astrocytes may reduce ferric iron to ferrous iron locally in the brain extracellular fluid, which can enable uptake and transport of NTBI by DMT1. Possibly discouraging this hypothesis, DMT1 in transfected astrocytes is not expressed in the cellular membrane but inside the cytosol (Pelizzoni et al., 2012). A second route may mediate uptake of ferric iron by a mechanism independent of transferrin receptors or ascorbate/DMT1 (Lane et al., 2010), and conditions with limitations in ascorbate may switch iron uptake to this route. Concurrent with this theory, DMT1 does not seem to co-localise with transferrin receptors in vesicles of cultured astrocytes (Pelizzoni et al., 2012). Inflammatory stimuli with TNF-α or IL-6 increase the expression of DMT1 in neurons, astrocytes, and microglia. They also induce the expression of hepcidin in astrocytes and microglia, but not in neurons (Urrutia et al., 2013).

### Future Perspectives for DMT1 Research

The importance of DMT1 for cellular iron uptake is evident, but many questions regarding its regulation remain. Insight into this highly complex regulation will increase the understanding of cellular iron uptake among neurons and glia and shed light on the treatment of diseases with altered iron metabolism. Vital questions on the understanding of iron transport at the BBB not only includes the role of DMT1, but also the roles of proteins like Steap and ferroportin for iron transport. Future studies should address these proteins and their roles for iron transport in experimental models of the BBB.

## Conflict of Interest Statement

The authors declare that the research was conducted in the absence of any commercial or financial relationships that could be construed as a potential conflict of interest.

## Footnotes

^1^www.enerca.org

## Abbreviations

BBB: blood-brain barrier
DMT1: divalent metal transporter 1
Fe^2+^: ferrous iron
Fe^3+^: ferric iron
IRE: iron response element
IRP: iron-responsive protein
NTBI: non-transferrin bound iron
TMD: transmembrane domain.
